# A New Dual-Input Deep Anomaly Detection Method for Early Faults Warning of Rolling Bearings

**DOI:** 10.3390/s23188013

**Published:** 2023-09-21

**Authors:** Yuxiang Kang, Guo Chen, Hao Wang, Wenping Pan, Xunkai Wei

**Affiliations:** 1 College of Civil Aviation, Nanjing University of Aeronautics and Astronautics, Nanjing 210016, China; kyxptt@nuaa.edu.cn (Y.K.);; 2 College of General Aviation and Flight, Nanjing University of Aeronautics and Astronautics, Nanjing 210016, China; 3 Beijing Aeronautical Engineering Technical Research Center, Beijing 100076, China

**Keywords:** rolling bearing, dual-input deep anomaly detection, unsupervised learning, zero fault samples, CNN

## Abstract

To address the problem of low fault diagnosis accuracy caused by insufficient fault samples of rolling bearings, a dual-input deep anomaly detection method with zero fault samples is proposed for early fault warning of rolling bearings. First, the main framework of dual-input feature extraction based on a convolutional neural network (CNN) is established, and the two outputs of the main frame are subjected to the autoencoder structure. Then, the secondary feature extraction is performed. At the same time, the experience pool structure is introduced to improve the feature learning ability of the network. A new objective loss function is also proposed to learn the network parameters. Then, the vibration acceleration signal is preprocessed by wavelet to obtain multiple signals in different frequency bands, and the two signals in the high-frequency band are two-dimensionally encoded and used as the network input. Finally, the unsupervised learning of the model is completed on five sets of actual full-life rolling bearing fault data sets relying only on some samples in a normal state. The verification results show that the proposed method can realize earlier than the RMS, Kurtosis, and other features. The early fault warning and the accuracy rate of more than 98% show that the method is highly capable of early fault warning and anomaly detection.

## 1. Introduction

As one of the universal and key components of rotating machinery, rolling bearings increase the cost of maintenance and cause unexpected accidents once failure occurs. Therefore, it is of great significance to explore more accurate, more efficient, and more intelligent early fault detection technology so that the monitoring of bearings can be realized in the early stage of bearing faults [1,2].

At present, with the development of artificial intelligence (AI) technology in the fields of image and voice [3,4], the technology has also been applied in the field of rolling bearing fault diagnosis [5]. In recent years, Deep Learning methods, such as CNN [6], Transfer Learning [7], and Deep Belief Networks (DBN) [8] have been widely used in the field of rolling bearing fault diagnosis and have obtained good diagnostic results. For example, Sun et al. [9] compressed the rolling bearing data by using stacked sparse autoencoders, thereby improving diagnostic accuracy. He et al. [10] realized the fault diagnosis of rolling bearings by using deep learning and acoustic emission technology. However, in actual engineering, it is difficult to obtain the operation data of the mechanical system in the ‘sick’ state. As a result, the obtained samples contain a large number of normal data and the abnormal data amount are very small or even zero in various typical fault states [11,12]. In this situation, the unsupervised anomaly detection (AD) method driven only by normal data has become an effective way of realizing the early AD of rolling bearings.

Classical AD methods such as the Support Vector Data Description (SVDD), Hyper-spherical Distance Discrimination (HDD), or PCA, were usually used for rolling bearing AD. Wu proposed a diagnosability analysis framework based on Deep PCA (Principal Component Analysis) and verified the effectiveness of the algorithm on the TDCS-FIB platform [13]. Wang [14] used Sparse Non-negative Matrix Factorization (SNMF) results as the input of SVDD, established a composite fault AD method for rolling bearings, and realized the accurate AD of composite faults of rolling bearings. Lin [15] proposed a novel HDD method to assess the performance of aero-engine bearings, which can solve problems with many parameters and high computational complexity. Liu [16] used PCA and the decision tree algorithm to realize the early fault warning of civil engine rolling bearings, with a warning rate up to 99.8%. However, Classical AD methods often require human experience to provide features and fail in high-dimensional samples [17].

In comparison, the AD method based on deep learning has also been applied in the early fault detection of rolling bearings. This kind of method mainly includes reconstruction-based methods and classification-based methods [18]. AE and generative adversarial networks (GAN) are representative of reconstruction-based methods. Huang [19] proposed a novel method, which can achieve 97.97% and 93.51% accuracy on the IMS and XJTU-SY datasets, respectively. Zhao [20] combined sparse autoencoder and transfer learning to propose a network model for rolling bearing AD. Wu [21] used the GAN + AE structure feature extraction network to realize rolling bearing AD. In addition, such Variational Autoencoder (VAE) [22] and many other reconstruction-based AD methods have achieved good performance in the early fault detection of rolling bearings. Deep Support Vector Data Description (DSVDD) [23] inherited the feature extraction advantages of deep learning and the classification performance of SVDD. Shao [24] used DSVDD for the early AD of rolling bearings and achieved good results. At the same time, Deep OC-NN [25] was also applied in the early AD of rolling bearings.

The evolution of rolling bearing spalling fault is an energy transfer process from high frequency to low frequency. In the early fault stage, the energy is mainly concentrated in the high-frequency band. In the middle stage of evolution, the energy is concentrated in the middle-frequency band. In the late stage of evolution, the energy is concentrated in the low-frequency band [26].

Traditional features such as root mean square value (RMS) and kurtosis value [19] characterize the evolution of this energy to a certain extent; that is, in the normal stage, such features tend to perform smoothly, and once bearings become abnormal, the value will change rapidly, through which the state of the bearing at this time can be judged. However, these features are easily affected by noise, working conditions, etc., resulting in irregular changes. If the AD method can extract a kind similar to the RMS or kurtosis, the feature not only reflects the ability to bear the failure of the energy transfer in the evolution process, making it more reliable and relative, but also, the differences in the normal and abnormal conditions are relatively more significant, and adopting the difference can be more intuitive to the identification of the abnormal state of bearing. This is very useful for achieving early fault detection of rolling bearings. Because of this, to extract this stable and reliable feature, and then realize the AD of bearing, this paper proposes a Dual-Input Deep Anomaly Detection (DIDAD) method by considering signals from different frequency bands. The method is applied to the early fault warning of rolling bearings, and the method is verified by using multiple sets of actual rolling bearing fault test data. The method is verified on multiple rolling bearing life data sets, which achieves better performance than the comparison methods.

The main innovations of this paper are:

1. A dual-input anomaly detection structure is proposed;
2. Introduce the experience pool structure into anomaly detection;
3. A new loss function is proposed.

## 2. Dual-Input Deep Anomaly Detection

### 2.1. The Overall Structure of DIDAD

**Figure 1:**
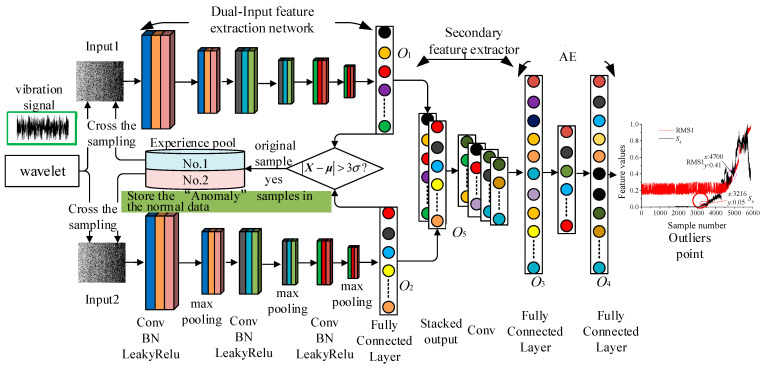
Dual-Input Deep Anomaly Detection.

The established DIDAD model is mainly composed of a dual-input feature extraction main framework, secondary feature extractor, autoencoder, experience pool, and other structures, and the specific structure is shown in Figure 1.

(1) Dual-Input feature extraction network based on a three-layer CNN. Firstly, wavelet decomposition is carried out to obtain signals of different frequency bands. Then, two high-frequency signals are selected as two inputs of the model. Finally, a three-layer convolutional neural network is used to extract features. The output results $o_{1}\in {\mathbb{R}}^{1\times m},o_{2}\in {\mathbb{R}}^{1\times m}$ of the two networks are laterally stacked and used as the input of the subsequent secondary feature extraction.
(2) The secondary feature extractor mainly uses a one-dimensional convolution kernel of size 5 × 1 to convolute the output $o_{5}$ (There are four outputs in this method. To facilitate writing, the four outputs are denoted as ***O***_1_, ***O***_2_, ***O***_3_, and ***O***_4_. ***O***_5_ is only an intermediate transition state and is not used to calculate the loss function.) to further extract features.
(3) AE is one of the classic unsupervised machine learning algorithms. In this paper, the AE module has three layers, the fully connected layer ***O***_3_, the encoder, and the decoder ***O***_4_. Among them, a one-dimensional convolution operation is used from the fully connected layer ***O***_3_ to the encoder, and a one-dimensional deconvolution operation is used between the encoder and the decoder ***O***_4_.
(4) DIDAD introduces the concept of experience pool. (The experience pool structure was proposed by the Google DeepMind team, and is mainly used to store state information data in reinforcement learning) [27]. For the batch samples of this training, the samples whose output results deviate from the overall mean value are stored in the designed experience pool cache. The balanced cross-sampling technique is used, and part samples and original data samples are randomly selected from the two experience pools to form a batch-size sample set as the input of the model in each training process.

In Figure 1: Conv is the convolutional layer [28], BN is a batch normalization layer [28], LeakyRelu is the activation layer, and the activation function is LeakyRelu [28].

NVIDIA GTX1660 6G is used in this experiment. i5-9600K CPU; the system is Windows 10; 8 GB of memory; and the programming language is python 3.7. The framework for all deep learning models is Pytorch 1.11; and the batch size is 128. The number of iterations is 200. The Adam optimization algorithm is used with a learning rate of 0.001.

### 2.2. A New Loss Function

For the training set with *n* samples, each sample has *p* features after passing through the convolutional network, assume that the ***X*** follows the normal distribution:

(1) $$X∼F_{p}(\mu ,\Sigma )$$

where $\mu ={\left(\mu_{1},\mu_{2},\mu_{3},\cdots ,\mu_{p}\right)}^{T}$ is the expectation of *X* and Σ is the covariance matrix. $F_{p}()$ is a normal distribution function.

The estimated value of and can be obtained via Equations (2) and (3)

(2) $$\tilde{\mu }=\frac{1}{n}\sum_{i=1}^{n}X_{i}$$

(3) $$\tilde{\Sigma }=\frac{1}{n}\sum_{i=1}^{n}(X_{i}-\tilde{u}){\left(X_{i}-\tilde{u}\right)}^{T}$$

For the four outputs ***O***_1_, ***O***_2_, ***O***_3_, and ***O***_4_ of DIDAD, Equations (2) and (3) can be used to calculate their corresponding global and with all data. They are recorded as global $\tilde{\kappa }=[{\tilde{\mu }}_{1},{\tilde{\mu }}_{2},{\tilde{\mu }}_{3},{\tilde{\mu }}_{4}],\tilde{\chi }=[{\tilde{\Sigma }}_{1},{\tilde{\Sigma }}_{2},{\tilde{\Sigma }}_{3},{\tilde{\Sigma }}_{4}]$. Similarly, if the batch size is *k* in the training process, the $\tilde{\mu }$ and $\tilde{\Sigma }$ of *k* samples obtained from the four outputs can be written as follows: $\bar{\kappa }=[{\bar{\mu }}_{1},{\bar{\mu }}_{2},{\bar{\mu }}_{3},{\bar{\mu }}_{4}],\bar{\chi }=[{\bar{\Sigma }}_{1},{\bar{\Sigma }}_{2},{\bar{\Sigma }}_{3},{\bar{\Sigma }}_{4}]$.

To make the output of *k* samples and the global computation have the same distribution, their corresponding expectation and covariance variance matrices need to be equal. The subloss function is proposed as shown in Equation (4):

(4) $$\begin{array}{l} {\tilde{L}}_{t}=\sum_{i=1}^{p}({\tilde{\kappa }}_{i}^{t}-{\bar{\kappa }}_{i}^{t}){}^{2}\\ \bar{L_{t}}=\sum_{j=1}^{p}\sum_{i=1}^{p}({\tilde{\chi }}_{ij}^{t}-{\bar{\chi }}_{ij}^{t}){}^{2} \end{array}$$

where ${\bar{L}}_{t}\;\mathrm{or}\;{\tilde{L}}_{t}$ are the *t*th (*t* = 1, 2, 3, 4), Covariance matrix error (CME) and expectation error (EE) of the outputs.

For the network driven only by normal data, the outputs ***O***_1_ and ***O***_2_ are the features that can denote the normal state. Therefore, the error between them should be as small as possible. For this reason, the error loss between these two outputs is considered:

(5) $$\begin{array}{l} {\tilde{L}}_{12}^{}=\sum_{i=1}^{p}{\left({\bar{\mu }}_{i}^{1}-{\bar{\mu }}_{i}^{2}\right)}^{2}\\ {\bar{L}}_{12}^{}=\sum_{j=1}^{p}\sum_{i=1}^{p}({\bar{\Sigma }}_{ij}^{1}-{\bar{\Sigma }}_{ij}^{2}){}^{2} \end{array}$$

In addition, the reconstruction errors between ***O***_3_ and ***O***_4_ are shown in Equation (6)

(6) $$\begin{array}{l} {\tilde{L}}_{34}^{}=\sum_{i=1}^{p}{\left({\bar{\mu }}_{i}^{3}-{\bar{\mu }}_{i}^{4}\right)}^{2}\\ {\bar{L}}_{34}^{}=\sum_{j=1}^{p}\sum_{i=1}^{p}{\left({\bar{\Sigma }}_{ij}^{3}-{\bar{\Sigma }}_{ij}^{4}\right)}^{2} \end{array}$$

All the subloss functions used to construct the joint loss function have been calculated at this point. The traditional joint loss function is to accumulate all subloss functions according to different weights, as shown in Equation (7)

(7) $$L=\sum_{z=1}^{T}\lambda_{z}L_{z}+\varepsilon \sum_{s=1}^{S}{\left\|w_{s}\right\|}^{2}$$

where *L* is the total loss, *T* is the number of subloss functions, $\lambda_{z}$ is the penalty factor of the *z*th subloss function $L_{z}$, $\varepsilon \sum_{s=1}^{S}{\left\|w_{s}\right\|}^{2}$ is the loss penalty term, ε is the penalty factor, ε = 10^−5^, *S* is the total number of network parameters, and *w_s_* is the *s*th network parameter.

One of the disadvantages of Equation (7) is that it is difficult to obtain the optimal $\lambda_{z}$ for each subloss function. In the calculation, it is assumed that every such subloss function has the same importance. Therefore, according to the proposed subloss function, this paper designs a joint loss function based on the max-min algorithm, as shown in Equation (8)

(8) $$L=\min\max([{\tilde{L}}_{t},{\bar{L}}_{t},{\tilde{L}}_{12}^{},{\bar{L}}_{12}^{},{\tilde{L}}_{34}^{},{\bar{L}}_{34}^{}])$$

Equation (8) firstly selects the maximum value of all the subloss values and then adopts the stochastic gradient descent algorithm to minimize the loss value. Equation (8) abandons the process that the traditional joint loss function needs to design the penalty factor, and only needs to optimize the maximum loss value in each training process. Theoretically, all the subloss functions will change toward their respective minimum values during the iteration process.

After the model’s training is completed, the anomaly score (the bearing fault evolution feature extracted in this paper) of all samples is calculated according to Equation (9), and the results are evaluated quantitatively via the AUC measure. For training, of course, we do not use any labels

(9) $$S_{t}=\log_{10}(\sum_{t=1}^{4}\sum_{i=1}^{p}({\tilde{\kappa }}_{i}^{t}-{\bar{\kappa }}_{i}^{t}){}^{2})$$

### 2.3. Experience Pool

The experience pool structure is mainly used to store the “Anomaly” samples in the normal data in the training process, and these “anomaly“ samples can participate in more training times by balanced cross-sampling, to achieve the purpose of improving the AD accuracy. 3σ criteria are mainly used to judge the “Anomaly” samples contained in the normal data.

(1) Compute the mean β of the *k* samples $X={\left(X_{i1},X_{i2},\cdots ,X_{ik}\right)}^{T}\begin{array}{cc} & i=1,2,\cdots ,p \end{array}$ in the batch and the mean ${\tilde{\mu }}_{r}$ and variance σ of the individual samples:

(10) $$\begin{array}{l} {\tilde{\mu }}_{r}=\frac{1}{p}\sum_{i=1}^{p}X_{ir}\\ \beta =\frac{1}{k}\sum_{r=1}^{k}{\tilde{\mu }}_{r}\\ \sigma =\frac{1}{k}\sum_{r=1}^{k}{\left({\tilde{\mu }}_{r}-\beta \right)}^{2} \end{array}$$

(2) Use Equation (11) to determine whether the current sample is an “anomaly”.

(11) $$\left|{\tilde{\mu }}_{r}-\beta \right|-3\sigma >0$$

(3) The samples of an “anomaly“ judged by Equation (11) are stored in the experience pool structure. (The anomaly here does not mean that it represents a fault, but a sample with poor performance in the normal state.)
(4) Balanced cross-sampling [26]. In the process of model training, part of the data are randomly selected from the experience pool and the original data set in proportion to form the batch samples required for the training, which are used as the input of the model. This ratio is set to 0.2, that is, 20% of the sample size of batch sizes is derived from the experience pool structure, and the remaining 80% is derived from the original dataset.

## 3. Examples of Early Fault Detection of Rolling Bearings

To verify the effectiveness of DIDAD in early fault warning of rolling bearings, four sets of run-to-failure experimental completed by the Intelligent Diagnosis and Expert System (IDES) laboratory of Nanjing University of Aeronautics and Astronautics and one set of run-to-failure experimental completed by the Intelligent Maintenance Systems (IMS) [27] Laboratory, University of Cincinnati, USA are studied using the proposed AD method. At the same time, the results are compared with DSVDD [23], DCGAN [23], ANOGAN [23], OC-NN [21] and other methods. In addition, to highlight the effect of the DIDAD on early fault warning, the DIDAD is compared with the RMS, Kurtosis, and other features that can characterize the fault.

### 3.1. Data Preprocessing

**Figure 2:**
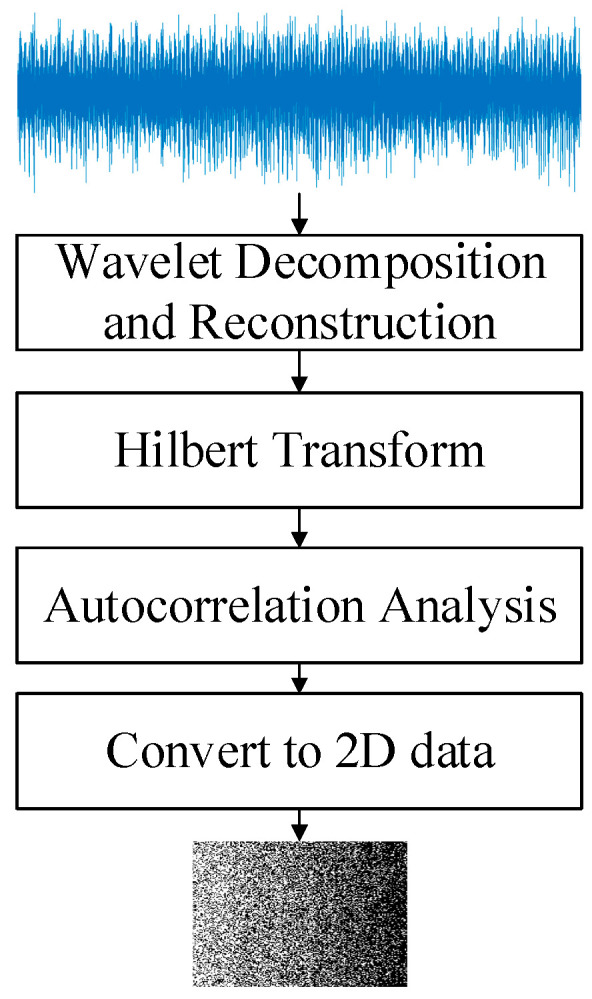
Rolling bearing vibration data preprocessing process.

All of the vibration signals are preprocessed in the same way. Wavelet decomposition and reconstruction methods are used to obtain data with m points in different frequency bands. The obtained sequence data are directly transformed into two-dimensional matrix data with c rows and d columns according to the column transformation, which is used as the input of the AD model. In this paper, the db8 [29] wavelet base is used to decompose the vibration signals of rolling bearings in five layers (This decomposition can be based on the characteristics of the signal and artificial experience, we have more experience usually decomposing the signal into five layers), and a total of five detail signals d1, d2, d3, d4, d5, and one approximate signal a5 are obtained. d1 and d2 signals are selected as the dual-inputs of the DIDAD model in this paper. The data preprocessing process is shown in Figure 2.

### 3.2. IDES Bearing Data Sets

**Figure 3:**
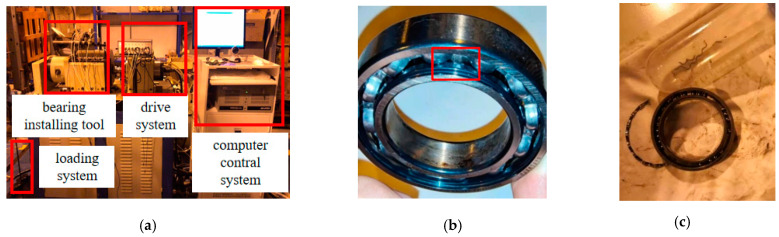
ABLT-1A bearing tester and fault bearing. (**a**) ABLT-1A test rig, (**b**) No. 2 bearing peel fault, (**c**) No. 3 bearing cage fault.

The IDES bearing data sets were collected from March to July 2021. It contains the damage evolution tests of 10 groups of bearings of 2 types. The parameters of the two types of bearings are shown in Table 1. The experimental system is ABLT-1A bearing strengthening testing machine developed by Hangzhou Bearing Test Center, as shown in Figure 3a. Four sets of bearings for a single test are installed in the test head, and four acceleration sensors are installed on the bearing housing to collect vibration acceleration signals of rolling bearings. The sampling frequency is 51,200 Hz, the sampling interval is 2.4 min, and the data volume of a single sample are 32,768 sampling points. What needs to be explained is that first, this is a test of rolling bearing in the whole life cycle; that is, the rolling bearing is intact at the beginning of the test, without any damage. After the test, No. 1 and No. 2 bearings are inner ring spalling faults, with a size of about 5 × 6 mm. Since No. 3 and No. 4 are cage fracture faults, the size of the fault cannot be calculated.

In the verification process, according to the location of the fault sample point in Table 1, the samples before the fault sample point are considered normal data, and the samples after the fault sample point are considered abnormal data.

In the process of model training, the first 1000 samples are used as normal samples for the BMD6009 bearing, and the first 500 samples are used as normal samples for the C&U61807 bearing. All samples are set as the testing set. As long as the model is trained according to the data of the normal state, the new data in the later stage are only used for input, and then the test results are given. There is no need to retrain in this process.

To illustrate the influence of the balance ratio on the results, 0.1, 0.2, 0.3 were selected for verification. The results are shown in Table 2. It can be seen from the results in Table 2 that when the selection ratio is 0.2, the optimal detection effect can be obtained on four sets of bearings.

The fault occurrence time points of various methods on the four test bearings and the AUC measure values are shown in Table 3.

The comparison results in Table 3 show that DIDAD can more accurately realize the early fault warning of bearings than other AD algorithms, and the accuracy on four rolling bearing test data sets reaches more than 99%. In the No. 3 bearing, DIDAD improves the accuracy by about 16.5% compared with the better-performing OC-NN. In the No. 4 bearing, the AUC value of DIDAD is 99.5%, while the value of ANOGAN is 94.8%. That is a relative increase of about 4.7%. In the No. 1 bearing, the AUC value of DIDAD is 99.8%, Compared with DSVDD, ANOGAN, and OC-NN, the results are improved by 2.5%, 2.9%, and 5.1%, respectively. In addition, the accuracy of DIDAD on the No.2 bearing is improved by 10.2%, 0.3%, and 0.5% compared with the other three models, respectively. The results show that the DIDAD shows good performances on AD with a high accuracy score in four bearing sets.

**Figure 4:**
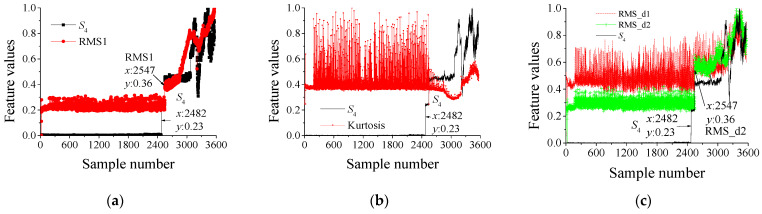

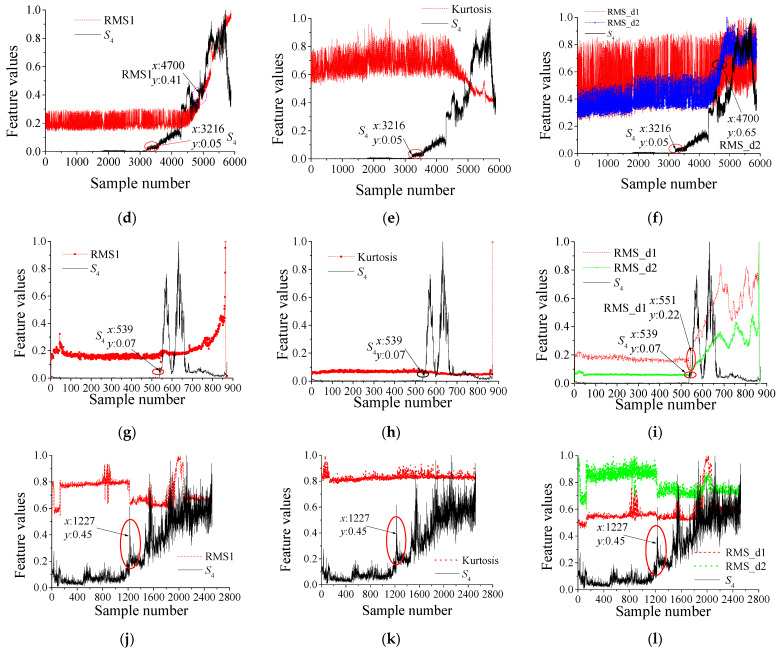
Multiple feature comparison results in the IDES dataset. (**a**) No. 1 bearing the *S*_4_ and RMS1, (**b**) No. 1 bearing the *S*_4_ and kurtosis, (**c**) No. 1 bearing the *S*_4_ and RMS_d1, RMS_d2, (**d**) No. 2 bearing the *S*_4_ and RMS1, (**e**) No. 2 bearing the *S*_4_ and kurtosis, (**f**) No. 2 bearing the *S*_4_ and RMS_d1, RMS_d2, (**g**) No. 3 bearing the *S*_4_ and RMS1, (**h**) No. 3 bearing the *S*_4_ and kurtosis, (**i**) No. 3 bearing the S4 and RMS_d1, RMS_d2, (**j**) No. 4 bearing the *S*_4_ and RMS1, (**k**) No. 4 bearing the *S*_4_ and kurtosis, (**l**) No. 4 bearing the *S*_4_ and RMS_d1, RMS_d2.

To further prove the superiority of the DIDAD, the score *S*_4_ of the output ***O***_4_ is selected as the evolution feature of the bearing and the RMS value (denoted as RMS1) and kurtosis in the full frequency band, as well as the RMS value (denoted as RMS_d1 and RMS_d2) of the d1 and d2 signals. The values of the above five features are very different in normal and abnormal states. Based on these differences, we can determine whether the current status is abnormal. The comparison results are shown in Figure 4. To visually compare and verify, each feature is divided by its maximum value in the process of drawing, to normalize the feature.

The comparison results show that, compared with the RMS1, kurtosis, and other features, the *S*_4_ is smoother in the whole life stage, and there is a significant difference between the normal and abnormal stages. Through this difference, the early abnormal state of bearings can be well judged. The more stable the *S*_4_ is in the normal stage, it shows that the vibration energy is stable at this time, and only in the normal stage can the vibration energy be stable. Therefore, in the normal stage, the smoother the *S*_4_ is, the better. When spalling occurs, the vibration energy will gradually increase with the size of the spalling, which is reflected in *S*_4_ as increasing or decreasing, which is completely different from the output value of the normal stage. If *S*_4_ is not smooth, fluctuations indicate that the vibration is unstable at this time. Therefore, it is difficult to distinguish between normal and fault states.

Specifically, the No. 1 bearing, RMS1 begins to show an increasing trend at the 2547th sample. However, the RMS_d1 and RMS_d2 fluctuated greatly during the whole test period, especially RMS_d1 could not even check its trend. In addition, the kurtosis value has no significant fault evolution trend. In contrast, in addition to the relatively smooth characteristics of S4, there is a significant increase before and after the failure. Compared with RMS1 finding the fault at the 2547th sample, DIDAD finds the early fault at the 2482 sample points, about 2.6 h earlier.

In the No. 2 bearing, the RMS_d1 and RMS_d2 fluctuate greatly during the whole test period; and RMS_d1 does not change at all. The RMS1, RMS_d2 and kurtosis values showed relatively significant changes at about the 4700th sample point, and S4 finds an anomaly at the 3216th sample, which is about 59 h earlier than that.

The No. 3 bearing has a small fluctuation in both RMS1 and kurtosis values. However, compared with S4, these two features only show an increasing trend in the late fault period. The RMS_d1 and RMS_d2 can identify the bearing anomaly at the 551th sample. However, the S4 is relatively more stable under the normal stage, and has a significant growth trend in the abnormal stage. The anomaly can be identified at the 539th sample about 0.5 h earlier.

RMS1, RMS_d1, RMS_d2 and the kurtosis value of the No. 4 bearing have no significant trend, which cannot directly reflect the fault state of the bearing. On the contrary, S4 shows a relatively more stable growth law. A spike occurred at point 1227, indicating an early bearing failure at this time.

Generally speaking, the value of the feature proposed in this paper is very stable under normal conditions, which is around zero, while the value of the abnormal state has particularly significant fluctuation, and the value is large. The results show that the features extracted by DIDAD can clearly distinguish the normal and abnormal states, are more sensitive than the RMS and kurtosis, and can identify the early fault earlier and more clearly.

### 3.3. IMS Bearing Data Sets

**Figure 5:**
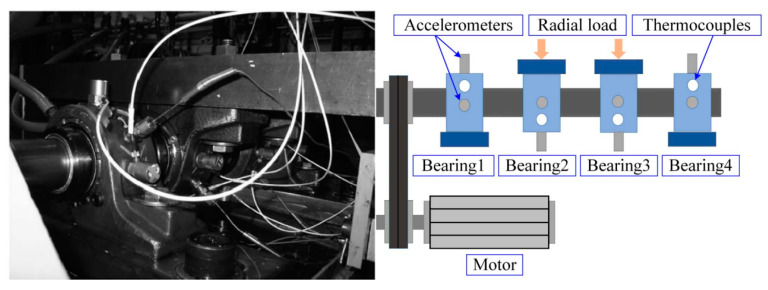
Rolling bearing life test bench of IMS.

To illustrate the versatility of this method and its advantages over traditional vibration features, we use the IMS data set for further verification. The IMS rolling bearing type is Rexnord ZA-2115 (Rexnord, Milwaukee, WI, USA). The experimental system is shown in Figure 5. The speed is constant at 2000 rpm, the sampling frequency is 20,480 Hz, the sampling points of each sample are 20,480, and the sampling interval is 10 min. The No.3 bearing in the test is used for AD test and verification. A total of 984 samples were collected during the bearing’s lifetime. According to the literature [19], the outer ring spalling fault occurred at the 533rd sample of this bearing. Therefore, in the process of AD, the first 300 samples are taken as the training set, and all the sample data are taken as the test set. In the test process, the larger AUC values calculated by d1 and d2 signals are taken as the AUC values. The test results are shown in Table 4.

The comparison results in Table 4 show that a DIDAD can accurately realize the early fault warning of bearings, and the warning accuracy can reach 100.0%. The accuracy of DSVDD, ANOGAN, and OC-NN are 99.9%, 100.0%, and 100.0%, respectively. Therefore, it is further proven that DIDAD can effectively realize the early fault warning of rolling bearings.

**Figure 6:**
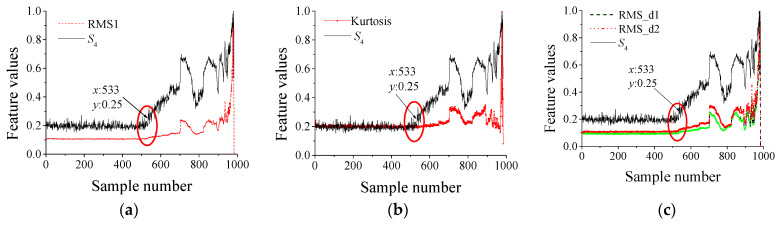
Multiple feature comparison results in IMS datasets. (**a**) The *S*_4_ and RMS1, (**b**) The *S*_4_ and kurtosis, (**c**) The *S*_4_ and RMS_d1, RMS_d2.

To further prove the superiority of the DIDAD, S4, RMS1, kurtosis, RMS_d1, and RMS_d2 are also selected for comparison verification. The comparison results are shown in Figure 6.

The results show that S4, RMS1, kurtosis, RMS_d1, and RMS_d2 are stable before the failure (533th sample). However, after the failure, the values of RMS1, kurtosis, RMS_d1 and RMS_d2 increase slowly, while S4 is steeper and the growth trend is more significant. Therefore, it can be seen intuitively that the features extracted by DIDAD are relatively more sensitive to normal and abnormal states, and the abnormal states of bearings can be identified earlier and clearer through it.

## 4. Discussion

The purpose of this paper is to propose a new fault detection method for rolling bearings. Starting from the data input of different frequency bands, we adopted a double-input anomaly detection method to verify the model on the rolling bearing life data set to demonstrate the effectiveness of the proposed method.

Compared with traditional methods, our proposed method has more advantages in anomaly detection, as shown in Table 2 and Table 3. We find that the trend of fault evolution of rolling bearings can be automatically extracted by using the double-input method, which is the same as the description of the trend of fault evolution of rolling bearings in the last section of the first section. There is little difference between a common IMS data set and traditional RMS and other feature values. However, in our experiments, the proposed method shows strong feature extraction capability. Compared with traditional eigenvalues such as RMS, the features extracted by this method have a more significant change trend.

In the future, it is still necessary to further verify the loss function and model structure of the model. The data of different frequency bands can be extracted directly from the spectrum as input, rather than the data obtained through the wavelet decomposition.

Finally, the proposed method can be further verified in practical work to verify its practicability in practical engineering problems.

## 5. Conclusions

A Dual-Input Deep Anomaly Detection (DIDAD) method is proposed in this paper. The loss function and structure of this method are introduced in detail. The whole life cycle tests of four rolling bearings are used for verification. The results show that compared with the traditional features such as RMS and kurtosis, the features obtained in DIDAD are more stable, and the differences between normal and abnormal stages are larger, which can be used to identify the early faults of rolling bearings more easily.

## Figures and Tables

**Table 1:** Rolling bearing parameter information.

Bearing Type		Inner Race/mm	Outer Race /mm	Thickness /mm	Rolling Element	Speed /rpm	Life /Hours	Sample Number	Fault Sample Point	Load (KN)		Failure Parts
										Radial	Axial	
BMD6009	No. 1	45	75	16	12	12,000	143	3564	2480	5.1	2.1	Inner
	No. 2						235.2	5884	3210	5.1	2.1	Inner
C&U 61807	No. 3	35	47	7	21	15,000	34.8	872	540	2.2	0	cage
	No. 4						101	2522	1220	2.2	0	cage

**Table 2:** Different ratios of test results.

Ratio	No. 1 Bearing	No. 2 Bearing	No. 3 Bearing	No. 4 Bearing
	AUC	AUC	AUC	AUC
0.1	98.9	99.8	99.4	99.5
0.2	99.8	99.9	99.7	99.5
0.3	99.4	99.5	98.9	98.8
0.4	98.3	99.7	99.1	98.2
0.5	98.1	99.1	97.9	97.6
0.6	97.9	99.9	98.2	97.1
0.7	98.4	99.7	97.5	98.2
0.8	98.8	99.2	97.9	98.3
0.9	98.6	99.1	98.3	97.7

**Table 3:** Test Results on the IDES dataset.

Method	No. 1 Bearing			No. 2 Bearing			No. 3 Bearing			No. 4 Bearing		
	d1	d2	AUC	d1	d2	AUC	d1	d2	AUC	d1	d2	AUC
DIDAD	2482		99.8	3216		99.9	539		99.7	1227		99.5
DSVDD	2506	2510	97.3	3627	3690	89.7	672	688	82.2	1463	1446	92.5
ANOGAN	2520	2515	96.9	3223	3657	99.6	669	685	83.1	1367	1389	94.8
OC-NN	2652	2652	94.7	3232	3248	99.4	665	691	83.2	1636	1649	89.6

**Table 4:** Test Results on the IMS Dataset.

Signal	DIDAD	DSVDD	ANOGAN	OC-NN
d1	533	534	533	533
d2		535	542	535
AUC	100.0	99.9	100.0	100.0

## Data Availability

Data sharing not applicable.
